# Breadsticks Flavoured with Olives and Onions: One-Year Shelf Life

**DOI:** 10.3390/foods12091798

**Published:** 2023-04-26

**Authors:** Angelo Maria Giuffrè, Manuela Caracciolo, Clotilde Zappia, Marco Capocasale, Marco Poiana

**Affiliations:** Dipartimento di Agraria, Università degli Studi Mediterranea di Reggio Calabria, 89124 Reggio Calabria, Italy; caracciolomanu@gmail.com (M.C.); czappia@outlook.it (C.Z.); marcocapocasale@outlook.it (M.C.); mpoiana@unirc.it (M.P.)

**Keywords:** baked products, breadstick, extra virgin olive oil, olive pomace oil, lipid oxidation, packaging, shelf life, Treccine

## Abstract

In this work, we compared breadsticks (known as *Treccine*) flavoured with onions and olives and prepared with olive pomace oil (OPO) or with extra virgin olive oil (EVOO). The effect on one-year shelf life was also studied. The following physical, chemical and sensory analyses were conducted on the breadsticks: water activity, moisture content, colour, texture and sensory analysis (appearance, colour, flavour, taste, texture and overall acceptability). For the oil extracted from the *Treccine*, we determined acidity, peroxide value, spectrophotometric parameters, ABTS and DPPH assay on the hydrolitic fraction, DPPH on the lipid fraction, and fatty acids. We detected a progressive deterioration in the quality of breadsticks with a decrease in shelf life after 4–6 months in relation to each studied parameter. In the analysed breadsticks, water activity was 0.342 (OPO recipe) and 0.387 (EVOO recipe) after one-year storage; in the same storage period, the moisture content was 6.34 times (OPO) and 5.32 times (EVOO) greater. Appearance and colour were the only two sensory parameters which, after 12 months, remained above or equal to five stated as the minimum quality value. In the extracted oil, Free acidity increased from 0.35 to 0.56% (OPO) and from 0.71 to 0.98% (EVOO); Peroxide value ranged between 6.10 and 102.89 meq/kg oil (OPO) and between 4.41 and 20.91 meq/kg oil (EVOO). K232 was highest in OPO (2.43–3.70) and lowest in EVOO (1.76–2.92), K268 was 1.32–1.580 (OPO recipe) and 0.570–0.640 (EVOO recipe). *Treccine* prepared with extra virgin olive oil showed better biological properties and longer shelf life.

## 1. Introduction

In recent decades, the food industry has focused on bakery products, both sweet [1,2,3,4,5,6,7,8,9,10,11,12,13,14] and savoury or similar bread products [15,16,17,18,19,20,21,22,23]. The bakery product market has changed together with modern society and the consequent objective of food industry to meet the consumer’s requests in relation to their hedonic and health expectations [24]. Modern life forces people to consume more ready-to-eat and takeaway foods. Breadsticks are suitable for this purpose because they have a pleasant taste and smell, come in individual portions, and are easily transported and eaten.

One of the most serious problems in food preparation is triglyceride hydrolysis, which is facilitated by food moisture. Another serious problem is fat oxidation, which is catalysed by high temperature, oxygen and light [25,26,27]. This oxidation occurs mainly due to the low antioxidant content of solvent extracted seed oil or olive pomace oil which are currently used in recipes for bakery products. Extra virgin olive oil (EVOO) is one of the main ingredients of the Mediterranean diet and its beneficial effects on the human health are well recognised [28]. Olive oil quality is influenced by many pre- and post-harvest factors [29], such as cultivar and harvest time [29,30,31,32,33,34,35]; harvest year [35,36]; climate change [36]; geographical area of production and age of trees [37]; extraction method [38,39]; storage conditions [40].

Breadsticks are Italian savoury products. They are dry snacks that can be flavoured in different ways and are also commonly used as a bread substitute. They can also be used as a snack.

**Figure 1:**
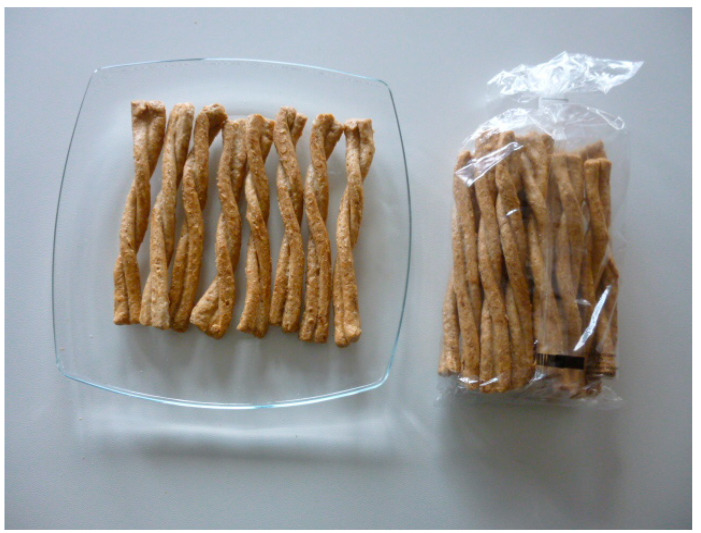
The breadsticks *Treccine*, flavoured with olives and onions.

Breadsticks prepared in Italy are usually single and have an elongated and cylindrical shape, while the breadsticks studied in our work (*Treccine*) were double, 20 cm long and with a twisted and plaited shape (Figure 1).

In many cases, the edible vegetable oil used in the recipe of bakery products is seed oil or low-quality olive oil, i.e., oil labelled as olive pomace oil (OPO) or olive oil. These olive categories are described by the Official International Regulations [41,42]. In our work, the *Treccine* recipe was modified by totally replacing OPO with EVOO produced in the Region of Calabria (Italy) with the aim of studying the lipid fraction evolution of *Treccine* flavoured with onions and olives.

## 2. Materials and Methods

### 2.1. Samples

The *Treccine* used in this experiment were industrially baked in the Region of Calabria (Italy), and they can be bought in supermarkets in Italy and abroad. We used some of the *Treccine* (*Treccine*, plural; *Treccina*, singular) prepared on a random day using the standardised production process.

The *Treccine* were prepared with the original formulation (control) with onions, olives and pomace olive oil and, at the same time, with a modified formulation with onions, olives and extra virgin olive oil. Both formulations were packaged (400 g per package) in transparent polypropylene film (PP) with air in the internal atmosphere. OPO and EVOO were obtained from Ottobratica olive cultivar which is autochthonous in the province of Reggio Calabria. EVOO was obtained by a three-phase extraction system. OPO was obtained from olive pomace after EVOO extraction. OPO was extracted with n-hexane. Except for the oil used, manufacturing conditions of the *Treccine* were the same for both recipes. A kilogramme of dough contained wheat flour 00 type (528 g); water (346 g); dehydrated onions (7 g); chopped black olives (7 g); oil (EVOO or OPO, 100 g); salt (NaCl 12 g). The *Treccine* were cooked at 200 °C for 10 min.

The storage conditions were similar to those implemented in a supermarket: room temperature (15–20 °C, according to season), with packages placed on a shelf, out of direct sun light. The *Treccine* physical–chemical–sensory characteristics of both OPO and EVOO recipes were studied, and analyses were conducted on the day of production (*t0*) and then after 2, 4, 6, 8, 10, 12 months (*t2*, *t4*, *t6*, *t8*, *t10* and *t12*, respectively). At each sampling, the lipid fraction was extracted to be analysed.

### 2.2. Analyses of Treccine

#### 2.2.1. Water Activity (a_w_)

Samples were ground and a_w_ was measured by a LabMaster-a_w_ Novasina instrument (Lachen, Switzerland). Analysis was conducted in six replicates: two replicates for each of three packages.

#### 2.2.2. Moisture Content Determination

Ground sample (10 g) was weighed and placed in an oven (105 °C) until constant weight was reached. Analysis was conducted in six replicates: two replicates for each of three packages.

#### 2.2.3. Textural Profile Analysis

Textural properties (hardness, g) of *Treccine* were evaluated by Three-point bending test (TPB) using a TA-XT plus Texture Analyser (Stable Micro Systems, Godalming, Surrey, UK). Each sample was placed on the two supports of the machine and a bar was moved vertically until it encountered the sample. Thus, the bar acts as a third contact point, exerting an increasing pressure until the structure of the product breaks. The maximum peak force was used to calculate the hardness value. The experiment was carried out following these conditions: pre-test speed: 1 mm·s^−1^, post-test speed: 15 mm·s^−1^, distance: 10 mm, test speed: 3 mm·s^−1^. Analysis was conducted in thirty replicates: two breakings on each of five *Treccine* for each of three packages.

#### 2.2.4. Colour Determination

Colour determination was conducted on the external surface and involved the measurement of L*, a*, b*, and C* parameters. A Konica Minolta colourimeter, model CM-A177 was used. Analysis was conducted in sixty replicates: four measures for each of five *Treccine* for each of three packages. The method and the calculation formula for Chroma (C*) and Whitness index are described in a previous work [2].

#### 2.2.5. Preliminary Sensory Analysis

The sensory semi-trained panel involved ten men and ten women aged to 19 to 60 years. The panellists were semi-trained on tasting *Treccine* and had previous experience with other food sensory tests. The panellists were asked to consider six attributes: colour, appearance, taste, flavour, texture and overall acceptability. Each attribute was evaluated on a 9-point hedonic scale (1–9) ranging from 1 (dislike extremely) to 9 (like extremely) for each characteristic and with 5 representing minimum quality. The test was conducted as described in a previous work for another bakery product (Cantuccini biscuits) [1] and as suggested by ‘Sensory Analysis—General Guidance for the Design of Test Rooms’ [43].

Colour (visual analysis) expressed the degree of toasting. Appearance defined the shape of the *Treccine*. Flavour and taste were related to the freshness of *Treccine*. Texture defined the crunchiness, and overall acceptability was the sum of the previous five attributes. The panellists were not informed about the ingredients of the *Treccine* before their assay, but before the test, they were asked if they had any food intolerance or allergy.

Each panellist received two *Treccine* each, one from two packages prepared with OPO, and the same was performed for EVOO recipe.

### 2.3. Oil Analysis

#### 2.3.1. Oil Extraction

The extraction of the lipid fraction was conducted as suggested by Folch et al. [44], with some modification. A total of 250 g of ground *Treccine* sample was weighed and homogenised with 1125 mL of a chloroform/methanol (2/1, *v*/*v*) solution at 40 °C for 20 min. The mixture was filtered through a 20–25 μm paper filter. To the filtrate, 750 mL of a 1 M potassium chloride (KCl) solution was added, and the mixture was left overnight at 4 °C to achieve phase separation. The lower phase, containing the lipid fraction, was collected, filtered through filter paper and anhydrous sodium sulphate (Na_2_SO_4_), and subsequently evaporated to dryness with a rotary vacuum evaporator.

#### 2.3.2. Free Acidity Determination

Free acidity was determined according to the European Commission [41]. The oil was dissolved in a diethyl ether/ethanol solution (1:1) and acidity was titrated with a 0.1 N NaOH solution with phenolptalein as an indicator.

#### 2.3.3. Peroxide Value Determination

The determination of peroxide value (PV) was carried out according to European Commission [41]. A total of 3 g of oil was weighed in a glass flask, with ground neck and stopper, and 10 mL of chloroform were used to dissolve the test portion rapidly by stirring. To this, 15 mL of acetic acid were added, and inert gas (N_2_) was used to remove oxygen. Next, 1 mL of a potassium iodide (KI) saturated solution was added, and the stopper was quickly inserted. After shaking for one minute, the sample was left for five minutes in the dark. At this point, 75 mL of deionised water and 6–7 drops of a 1% starch solution were added as indicators. The determination was carried out by titrating the liberated iodine with the 0.01 N sodium thiosulphate (Na_2_S_2_O_3_) solution, shaking vigorously.

The peroxide value (PV), expressed in milliequivalents of active oxygen per kilogram of oil (meq O_2_·kg^−1^), is expressed by the formula proposed by the European Commission [41].

#### 2.3.4. Spectrophotometric Investigation in Ultraviolet

Spectrophotometric investigation in ultraviolet was carried out to obtain information about the oxidative condition of the oil at each sampling time. The determination of extinction coefficients (K_232_ and K_268_) was carried out according to European Commission [41]. An UV/Vis Spectrometer λ2, Perkin Elmer (Waltham, MA, USA), was used.

#### 2.3.5. Antioxidant Capacity of the Extracted Oil

Antioxidant capacity was studied both on Hydrophilic Antioxidant Extract (HAE) and also directly on the oil. HAE was obtained following the method proposed by Goldsmith et al. [45] with the following modifications. A 2.5 g aliquot of each sample was mixed for extraction with 5 mL of methanol/water solution (80/20, *v*/*v*). After shaking with a Vortex for 1 min, the mixture was centrifuged at 5000 rpm for 7 min. The supernatant containing the antioxidants was kept. This procedure was repeated once more by adding 5 mL of methanol/water solution and the two extracts were mixed together and analysed.

#### 2.3.6. ABTS Assay on Hydrophilic Antioxidant Extract

The scavenging activity of HAE was determined with the method proposed by Re et al. [46] with some modifications. A 0.050 mL aliquot of HAE was added to 2.450 mL of a 7 mM ABTS ethanolic solution and the absorbance was immediately measured (*abs t0*) at 734 nm using an Agilent 8453 spectrophotometer (Santa Clara, CA, USA). After measuring, the mixture was vigorously shaken in the dark for 6 min. Then, the absorbance was again measured (*abs t6*). The radical scavenging activity was calculated as % of inhibition [47]. The radical scavenging activity (% of inhibition) was plotted against a Trolox calibration curve and results were then expressed as TEAC values (μmol TE∙100 g^−1^ of fat extracted).

#### 2.3.7. DPPH Assay on Hydrophilic Antioxidant Extract

The antioxidant capacity was determined following the method proposed by Kalantzakis et al. [47] and the following modifications were applied.

A 0.10 mL aliquot of HAE was added to 2.40 mL of a 60 µM DPPH methanolic solution and the absorbance was immediately measured (*abs t0*) at 515 nm using an Agilent 8453 spectrophotometer (Santa Clara, CA, USA). After measuring, the mixture was vigorously shaken in the dark for 5 min. Then, the absorbance was again measured (*abs t5*). The radical scavenging activity was calculated as % of inhibition [47]. The radical scavenging activity (% of inhibition) was plotted against a Trolox calibration curve and results were then expressed as TEAC values (μmol TE∙100 g^−1^ of fat extracted).

#### 2.3.8. DPPH Assay on the Extracted Oil

The DPPH assay was also performed directly on the extracted oil as raw material. The determination was conducted in an UV/Vis Spectrometer λ2, Perkin Elmer (Waltham, MA, USA) using the method proposed by Kalantzakis et al. [47], modified as follows. First of all, the oil was diluted with Ethyl Acetate (1/10, *v*/*v*). Then, 0.5 mL of diluted oil was added to 2 mL of a 10^−4^ M DPPH^●^ solution, previously prepared with ethyl acetate. Following this, the absorbance of the mixture was immediately measured at 515 nm (*abs t0*) and after 30 min of shaking and incubation in the dark (*abs t30*). The % of inhibition was calculated as described by [48]. The radical scavenging activity (% of inhibition) was compared with a Trolox calibration curve and results were then expressed as TEAC values (μmol TE∙100 g^−1^ of fat extracted).

#### 2.3.9. Colour of the Extracted Oil

Colour was instrumentally determined as described in a previous work [48]. A Minolta Chroma Meter CR-400 instrument was used, equipped with a transparent (base and side) special, cylindrical glass container (5.0 cm, 6.0 cm high). The glass container was filled with one centimetre of extracted oil and the colour was evaluated. The CIELab scale was used. L* (brightness) ranged between 0 (black) and 100 (white); a* ranged between −90 (green) and +90 (red) and b* ranged between −90 (blue) and +90 (yellow).

#### 2.3.10. Determination of Fatty Acids by Gas Chromatography

Fatty acids were analysed by conversion into their methyl esters according to the European Commission [41]. Fatty acid methyl esters (FAMEs) were analysed by gas chromatography (Thermo Trace 1300, Thermo Fisher Scientific, Waltham, MA, USA) fitted with a flame-ionisation detector (FID) and split injector. The temperature of the split injector was 250 °C, with a split ratio of 35, and the detector temperature was 280 °C. A capillary column SUPELCOWAXth–10 with 30 m length × 0.32 mm i.d., and 0.5 ìm film thickness was used. The oven temperature was 100 °C; then, it was increased up to 160 °C, at 2 °C∙min^−1^; then, it was isothermal at 160 °C for 5 min, and then increased to 230 °C at 4 °C∙min^−1^ and finally held at 230 °C for 10 min. Helium, air and hydrogen flows were, respectively, 2.7 mL∙min^−1^, 350 mL∙min^−1^ and 35 mL∙min^−1^, kept constant overtime. FAMEs were identified by comparing their retention times with those of pure standards previously injected and with the literature data. Results were expressed as % m/m.

#### 2.3.11. Statistical Analysis

Analysis in oil for free acidity, peroxide value, spectrophotometric indices, antioxidant capacity, colour and fatty acids was conducted in triplicates: one replicate for each of three packages. The replicates of each analysis are specified in the section describing the analytical method. Data were subjected to analysis of variance (one-way ANOVA, post hoc Tukey test *p* < 0.05 using IBM SPSS Statistics 25.0 Software (IBM, Armonk, NY, USA)). Values are expressed as means ± SD. Different small letters indicate significant differences among the different times of storage (*, *p* < 0.05; **, *p* < 0.01; ***, *p* < 0.001; n.s., *p* > 0.05).

## 3. Results

### 3.1. Water Activity and Moisture Content

**Figure 2:**
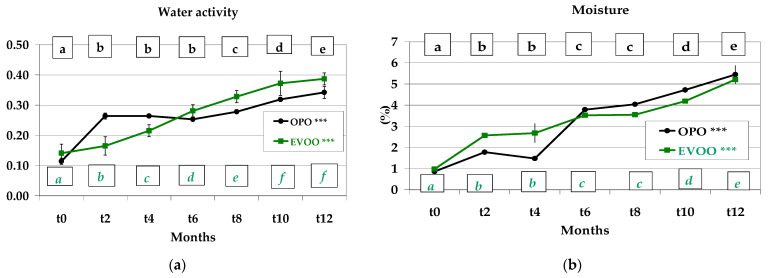
(**a**) Water activity of *Treccine* prepared with olive pomace oil and with extra virgin olive oil during one-year shelf life; (**b**) moisture of *Treccine* prepared with olive pomace oil and with extra virgin olive oil during one-year shelf life. The green line and green letters are related to *Treccine* prepared with EVOO. The black line and black letters are related to *Treccine* prepared with OPO. Values with different letters with the same colour are significantly different: *** *p* < 0.001.

Water activity increased significantly (*p* < 0.001) during the 12-month storage in both EVOO (0.141–0.387) and OPO (0.114–0.342) *Treccine*. Over one-year period, a_w_ in the OPO recipe increased 3-fold, whereas in the EVOO recipe the increase was determined to be 2.74-fold. *Treccine* prepared with OPO showed the highest values at *t10* and *t12* (0.319 and 0.342, respectively); also, EVOO showed the highest a_w_ at *t10* and *t12* but with highest values 0.372 and 0.387, respectively (Figure 2a). These findings showed a better ability of OPO to contrast the increase in a_w_. In addition, moisture content showed a significant (*p* < 0.001) and continuous increase during storage in both formulations. In detail, it ranged between 0.86 (*t0*) and 5.45% (*t12*) in OPO *Treccine* (i.e., a 634% increase) and between 0.98 (*t0*) and 5.21% (*t12*) (i.e., a 532% increase) in EVOO *Treccine* (Figure 2b). This behaviour can be explained because a plastic film is not an impenetrable barrier: gases and aqueous vapour can permeate this film and permeability is related to temperature, relative humidity and pressure [49,50]. Furthermore, we did not modify the process applied by the *Treccine* industry, and the original headspace of the package used in this experiment was not modified by introducing inert gas such as N2.

Conte et al. [51] evidenced the effect of olive leaf water extract and phenolic olive mill wastewater extract on both moisture content and water activity and that the addition of these extracts caused an increase in moisture content and water activity compared to the control in gluten-free breadsticks.

Alamprese et al. [52] studied breadsticks prepared with EVOO, packaged (125 g each pack) in a transparent PP film under air and stored at 20 °C in which the moisture content value during 200-day storage increased from 2.09 to 5% with a double initial value and a very similar final content compared to our samples.

Jemziya and Mahendran [53] studied 12-month-stored cookies produced with a blended flour (potato flour and wheat flour) packed in sealed laminate aluminum foil and determined that the continuous moisture content increase in cookies was influenced by the potato flour in the recipe: the higher the potato flour percentage, the higher the moisture content increase during storage.

### 3.2. Hardness

**Figure 3:**
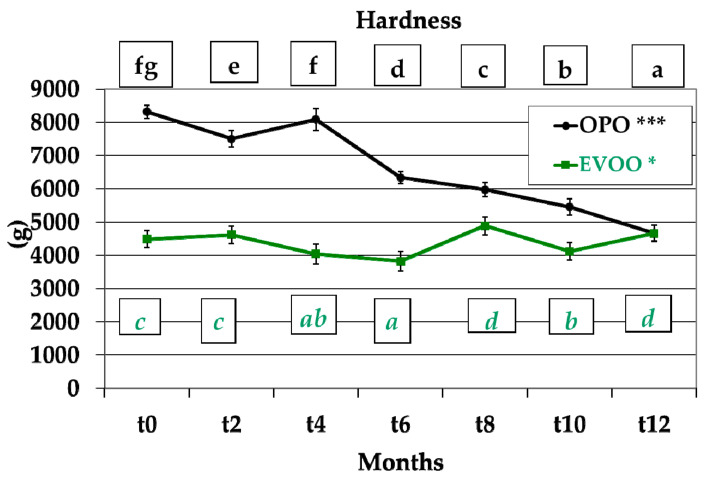
Hardness of *Treccine* prepared with olive pomace oil and with extra virgin olive oil during one-year shelf life. The green line and green letters are related to *Treccine* prepared with EVOO. The black line and black letters are related to *Treccine* prepared with OPO. Values with different letters with the same colour are significantly different: *** *p* < 0.001; * *p* < 0.05.

*Treccine* prepared with the two studied formulations showed two different hardness behaviours. Those with OPO in the recipe showed very high significant differences (*p* < 0.001) during storage with a decreasing trend from 8317 (*t0*) to 4671 (*t12*) (Figure 3). Those prepared with EVOO showed significant differences (*p* < 0.05) during storage from *t0* (4484) to *t12* (4662). At the same time, it was verified that at each sampling, *Treccine* with EVOO formulation had a lower hardness value than *Treccine* with OPO formulation. Hardness variation during *Treccine* storage can be related to the permeability of the packaging and the flowing, during storage, of the external atmosphere with aqueous vapor in the package. It can also be due to the aging process of the food, including interaction between molecules, starch retrogradation and moisture migration [54,55]. The difference in hardness values between the two different recipes could be due to the different oil composition [56,57] and its interaction with the ingredients of *Treccine* breadsticks.

Many authors have studied the variation in hardness in breadsticks; their recipes were modified to improve the biological properties of breadsticks. Conte et al. [51] studied the hardness in relation with the variation of the original breadstick recipe by adding phenolic-rich extracts from olive leaves and olive mill wastewater in different quantities and determined that the original recipe showed a higher hardness compared with the modified recipe, irrespective of the quantity of the added phenolic extracts.

Cox and Abu-Ghannam [58] studied the variation in hardness in relation to the incorporation of *Himanthalia elongata* seaweed and determined that the higher the flour fortification with seaweed, the higher the harness in breadsticks.

De Gennaro et al. [59] partially replaced maize flour with lyophilised olive cake powder and determined that the higher the content of lyophilised olive cake powder, the lower the hardness in breadsticks whose original recipe (control) contained rice flour (41%), water (35%), sunflower oil (11%), maize flour (9%) psyllium fibre (2%), baking powder (1.5%; disodium diphosphate, sodium hydrogen carbonate, maize starch), and salt (0.5%).

### 3.3. Colour, Instrumental Analysis

Colour is a very important parameter because it highly influences the consumer’s preference, in particular when the packaging is transparent and it is easy to verify the contents. Only these parameters can be evaluated by customers prior to opening of the package, and customers relate the colour of a bakery product such as breadsticks to attributes such as crispness or being under- or overcooked.

**Figure 4:**
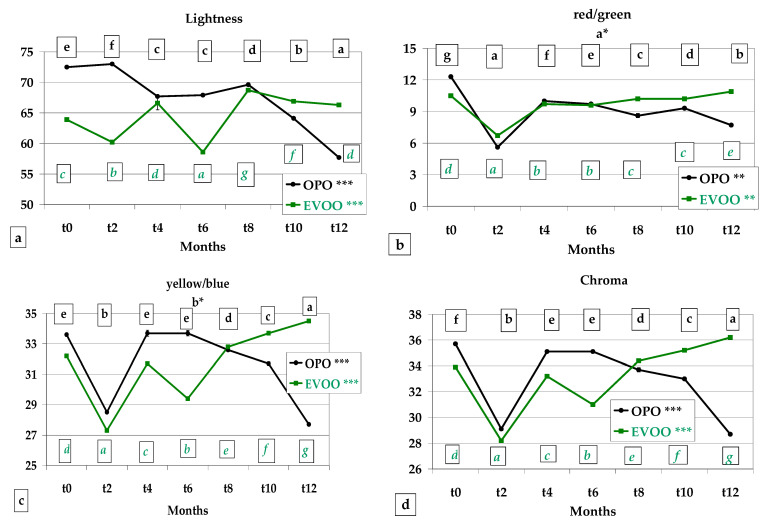

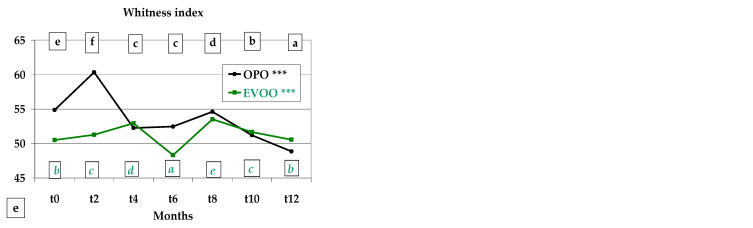
Colour by instrumental analysis of *Treccine* prepared with olive pomace oil and with extra virgin olive oil during one-year shelf life. (**a**) Lightness; (**b**) red/green; (**c**) yellow/blue; (**d**) Chroma; (**e**) Whitness index. The green line and green letters are related to *Treccine* prepared with EVOO. The black line and black letters are related to *Treccine* prepared with OPO. Values with different letters with the same colour are significantly different *** *p* < 0.001; ** *p* < 0.01.

Lightness (L*) was highest in OPO *Treccine* until *t8*, whereas the EVOO values prevailed in the last four months storage (*p* < 0.001 in both recipes) (Figure 4a).

The a* values showed a parallel behaviour during storage with an initial fall from *t0* to *t2*, an increase from *t2* to *t4* and a non-significant variation until *t12* (*p* < 0.01 in both recipes) (Figure 4b). The b* values proceeded similarly for the first 6 months of storage, after which, the OPO *Treccine* values constantly decreased until *t12* from 33.70 to 27.70. On the contrary, the EVOO values increased from 29.40 to 34.50 (Figure 4c). Chroma evidenced a similar trend with b* (Figure 4d). Whiteness of EVOO *Treccine* was determined to be more stable during storage, varying in a range from 48.32 to 53.53 (*p* < 0.001), whereas in OPO *Treccine*, the range was 48.85–60.34 (*p* < 0.001) (Figure 4e). The difference in colour of the two types of oil and consequently of the two *Treccine* was due to the refining process applied to OPO after extraction from olive pomace; in fact, this process, on the one hand, lowers free acidity and eliminates any oxidised substances, but on the other, lowers pigment content and influences the colour.

Rakshit et al. [60] studied the 98-day shelf life of biscuits prepared with pomegranate peel extract and sunflower oil and reported a non-significant change in L*, a* and b*. In a prolonged study (12 months) on *Cantuccini* biscuits prepared with margarine: butter (50:50) and with butter: EVOO (30:70), the authors reported a significant variation L*a*b* during storage, with a tendency to decrease in L* and b* and an increase in a* [1].

### 3.4. Sensory Analysis

**Figure 5:**
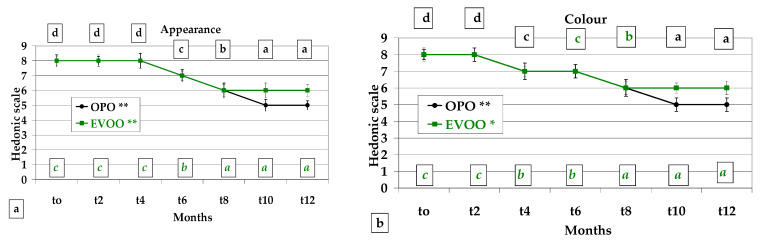

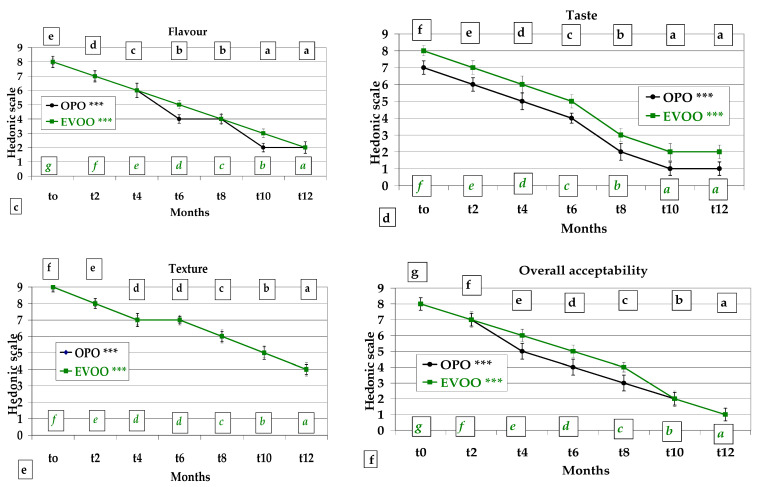
Sensory analysis of *Treccine* prepared with olive pomace oil and with extra virgin olive oil during one-year shelf life. (**a**) Appearance; (**b**) colour; (**c**) flavour; (**d**) taste; (**e**) texture; (**f**) overall acceptability. The green line and green letters are related to *Treccine* prepared with EVOO. The black line and black letters are related to *Treccine* prepared with OPO. Values with different letters with the same colour are significantly different: *** *p* < 0.001; ** *p* < 0.01; * *p* < 0.05.

Appearance showed the same trend as colour, even if at *t4* the panellists assigned eight points instead of seven assigned to colour at the same sampling (*p* < 0.01 for both OPO and EVOO during 12-month storage) (Figure 5a).

Colour was not influenced by the type of oil in the first 8 months of storage, whereas a preference for *Treccine* prepared with EVOO was detected at *t10* and *t12*. Five points (minimum quality) were attributed only to OPO *Treccine* at *t10* and *t12.* Colour was significantly influenced during storage (*p* < 0.01 OPO recipe and *p* < 0.05 EVOO recipe) (Figure 5b).

Flavour evaluation showed a similar decreasing trend in both recipes for the first 4 months of storage, from 8 (*t0*) to 6 points (*t4*), and reached the minimum quality score (i.e., 5) at *t6* for EVOO (*p* < 0.001), whereas the *Treccine* prepared with OPO were below the minimum quality score after *t6* (*p* < 0.001 during one year storage). Flavour and aroma are partially dependent on the type of oil and volatiles contained in EVOO. In addition, antioxidants contained in EVOO contrasted and reduced oil oxidation and the production of a rancid odour (Figure 5c).

Taste was determined to be influenced by both olive oil type and storage. The *Treccine* prepared with EVOO were always preferred to the ones prepared with OPO, which was dependent on the volatiles contained in EVOO influencing the positive notes of flavour which disappear during OPO refining. The EVOO *Treccine* obtained the minimum taste quality after 6-month storage (*p* < 0.001 in one year), whereas OPO *Treccine* were considered acceptable for 4-month’s storage (*p* < 0.001 in one year). In both cases, in the last 6-months of shelf life, the taste quality decreased rapidly and was far from 5 as minimum quality mainly for OPO recipe (Figure 5d).

Texture was not influenced by the type of the oil but only by shelf life. *Treccine* were evaluated with the maximum score (*t0*) and with eight and seven points, respectively, at *t2* and *t4–t6*. The minimum quality texture was at *t10*, whereas at *t12* the texture (scored with four points) was judged below the minimum. Texture was significantly influenced during storage (*p* < 0.001 OPO recipe and *p* < 0.001 EVOO recipe) (Figure 5e).

Overall acceptability was highest (eight points) at *t0* and decreased during storage. The minimum quality value for overall acceptability was at *t4* for OPO recipe and at *t6* for EVOO recipe. This decreasing trend was influenced by the natural oxidation of both types of oils (with a preference for EVOO) (*p* < 0.001) and by the presence of onion whose fast degradation during storage influenced mainly taste and flavour (Figure 5f).

**Figure 6:**
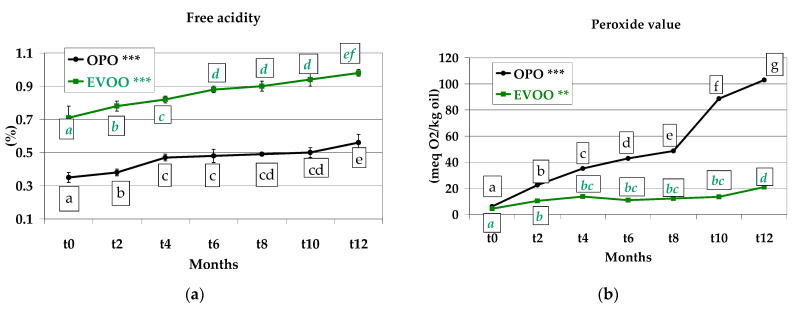
(**a**) Free acidity and (**b**) peroxide value of olive pomace oil and extra virgin olive oil extracted from *Treccine* during one-year shelf life. The green line and green letters are related to *Treccine* prepared with EVOO. The black line and black letters are related to *Treccine* prepared with OPO. Values with different letters with the same colour are significantly different: *** *p* < 0.001; ** *p* < 0.01.

OPO and EVOO slightly influenced the attributes related to visual stimulus, probably because browning due to baking similarly influenced the visual appreciation of colour and appearance. In addition, Texture was determined not to be influenced by the type of olive oil. It is noteworthy that EVOO influenced the Taste score throughout the whole year. EVOO positively influenced the Flavour score in the second part of the storage period (*t4*–*t12*), which is due to the phenolic content of EVOO and the reduced oxidation rate with a consequent low production of characteristic unpleasant odour. This must be related to the behaviour of the peroxide value (Figure 6b).

Ktenioudaki et al. [61] studied the hardness of breadsticks prepared without oil or fat and stored for 91 days in a plastic bag. They detected a decreasing tendency of the hardness value, with a different rate in relation to the recipe and with breadsticks prepared with 100% wheat flour showing the most evident variation.

Hammad et al. [62] studied breadsticks fortified with Grinko and Ginseng and reported an inverse correlation between oxidative indices (peroxide value, *p*-anisidine value, totox) and sensory parameters such as odour, taste and texture. This agrees with our results.

### 3.5. Free Acidity and Peroxide Value of the Extracted Oils

Free acidity and peroxide values are two of the most important parameters to evaluate the chemical quality of olive oil because they describe the condition of two dangerous alterations: acidification and oxidation. Testing these parameters is both cheap and easy. Free acidity is due to the hydrolysation of tryglycerides with a consequent separation of fatty acids from a triglyceride; they remain free in olive oil. This reaction is facilitated by the moisture. Free acidity in OPO extracted from *Treccine* increased significantly (*p* < 0.001) from *t0* (0.35%) to *t12* (0.56%), showing a 1.56-fold increase, whereas in the oil of EVOO recipe, free acidity ranged between 0.71% (*t0*) and 0.98% (*t12*), with a 1.38-fold increase (Figure 6a). The free acidity of both OPO and EVOO increased constantly during storage. The low free acidity of OPO was due to its rectification before usage in the food industry, which is necessary to obtain edible oil. This value, lower than EVOO, was maintained during storage. A study was conducted on Italian Cantuccini biscuits during one-year storage, in which fat content was 70% EVOO and 30% cow butter; here, an increase in free acidity from 1.02% to 1.35% was detected, i.e., a 1.32-fold increase, similar to that of EVOO *Treccine* prepared with onions.

Oxidation occurs mainly due to the high temperature of storage and the presence of light and oxygen inside the package. At each sampling, the peroxide value was lowest in olive oil from the EVOO recipe, ranging between 4.41 meq O_2_/kg oil (*t0*) and 20.91 meq O_2_/kg oil (*t12*). This means a 4.74-fold increase in one year (*p* < 0.01). This occurred due to the antioxidants existing in EVOO which combat the oil oxidation. It is noteworthy that the maximum value reached after one-year storage in EVOO recipe (20.91 meq O_2_/kg oil) was slightly higher than the maximum value (20 meq O_2_/kg oil) indicated by the European legislation [41] and by International Olive Council [42] for an extra virgin olive oil. A completely different behaviour was detected in the oil extracted from the OPO recipe, which showed a fast and high oxidation from 6.10 meq O_2_/kg oil (*t0*) to 102.89 meq O_2_/kg oil (*t12*) (*p* < 0.001). In detail, in OPO recipe-extracted oil, the oxidation more than tripled in the first two months of storage and showed a 16.87-fold increase after one year of storage, displaying no attitude to oxidative resistance and a fast and strong deterioration of the OPO and consequently a fast deterioration of the *Treccine* prepared with this type of oil **(**Figure 6b). The OPO fast oxidation and its unpleasant odour was observed by panellists during the sensory analysis of Flavour (Figure 5c) and mainly of Taste (Figure 5d), also showing that oxidation was the most notable problem during storage in a *Treccine* flavoured with OPO and with EVOO.

In studies conducted on *Treccine* prepared with sunflower oil and fortified with vegetable matrices, it was determined that Ginkgo and Ginseng significantly reduced the PV increase during 60-day storage up to a maximum of 9 meq O_2_/kg oil in relation to the percentage of extract included in the recipe (the higher the extract content, the lower the PV increase), whereas in the control sample (without vegetable extract), the PV increased up to 15 meq O_2_/kg oil in 25-day storage [62]. Caponio et al. [63] studied the peroxide value evolution in a traditional bakery product called *Taralli* prepared in Apulia (Italy) with different oils (extra virgin olive oil, olive oil, olive pomace oil and refined palm oil) and detected an increase in PV in all different recipes even though EVOO showed the lowest significant increase.

### 3.6. Spectrophotometric Indices of the Extracted Oils

**Figure 7:**
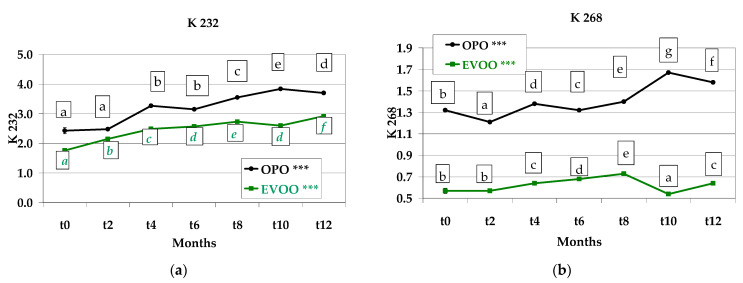
(**a**) K 232 and (**b**) K 268 values of olive pomace oil and extra virgin olive oil extracted from *Treccine* during one-year shelf life. The green line and green letters are related to *Treccine* prepared with EVOO. The black line and black letters are related to *Treccine* prepared with OPO. Values with different letters with the same colour are significantly different: *** *p* < 0.001.

K232 indicates the presence of conjugated dienes and the initial state of the oxidation in an oil or fat. As expected, the K232 values were lowest in the oil extracted from *Treccine* with the EVOO recipe and ranged between 1.76 (*t0*) and 2.92 (*t12*), namely displaying a 166% constant and significant (*p* < 0.001) increase. The values of oil from the OPO recipe increased from 2.43 (*t0*) to 3.70 (*t12*), namely displaying a 152% significant (*p* < 0.001) increase. At each sampling, the K232 value of OPO recipe was higher than that of the EVOO recipe, showing the lower oxidative values of EVOO recipe maintained during storage (Figure 7a).

K268 indicates the presence of conjugated trienes and a pronounced state of oxidation. At each sampling, the oil obtained from the OPO recipe showed a value more than double compared to the oil obtained from EVOO recipe. In detail, K_268_ of EVOO recipe was lowest at *t0* (0.57) and showed a tendency to increase until *t8* (0.73) and subsequently decrease at *t10* (0.54) and *t12* (0.64). K268 of OPO recipe showed a tendency to increase from *t0* (1.32) and *t10* (1.67) and slightly decrease at *t12* (1.58). In addition, in this case, better oxidative properties of EVOO recipe were evidenced (Figure 7b). Both K232 and K268 values increased with the oil refining process.

In a study conducted on EVOO and OPO oils, it was determined that both the heating duration (30–60–120 min) and the applied temperatures (180–200 °C) influenced the spectrophotometric indices and that the highest temperature (220 °C) caused more oxidative damage than the heat exposure time [25]. The spectrophotometric indices were determined to increase with storage in the lipidic fraction of a traditional Italian bakery product (*Taralli*) prepared with different oils and stored for five months. Similarly to our results, the indices of EVOO were lower than those of OPO, both for diene and for triene conjugates [35].

### 3.7. Antioxidant Capacity

**Figure 8:**
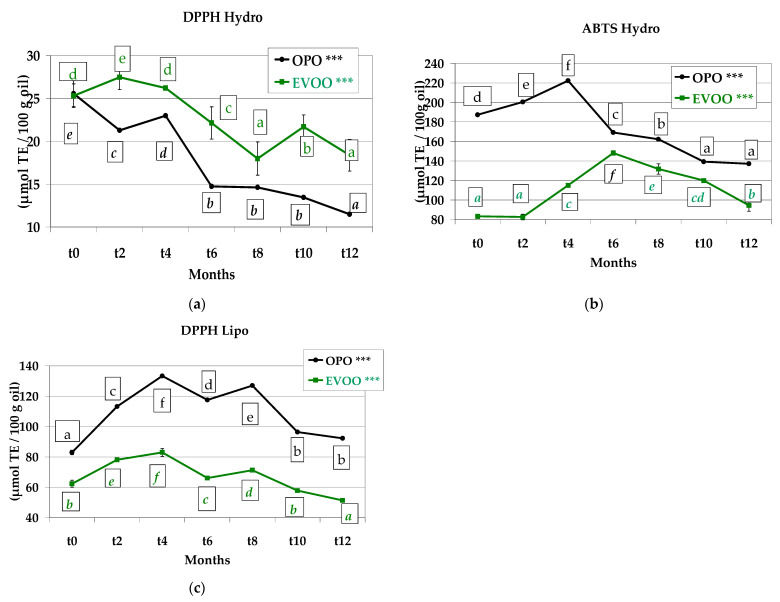
(**a**) DPPH hydrophilic fraction, (**b**) ABTS hydrophilic fraction, (**c**) DPPH lipolytic fraction, of olive pomace oil and extra virgin olive oil extracted from *Treccine* during one-year shelf life. The green line and green letters are related to *Treccine* prepared with EVOO. The black line and black letters are related to *Treccine* prepared with OPO. Values with different letters with the same colour are significantly different: *** *p* < 0.001.

The antioxidant capacity was studied on the extracted oil and on the hydrophilic fraction obtained from the oil (Figure 8a–c). DPPH hydro values showed a tendency to decrease during shelf life (Figure 8a). In the ABTS hydro assay, all values increased for both recipes with storage duration from *t0* to *t4* (OPO) and to *t6* (EVOO), and then started to decrease (Figure 8b). The antioxidant capacity may be overestimated because ABTS^•+^ reacts with any hydroxylated aromatics independently of their real antioxidative potential. In fact, the ABTS test is reduced to titration of aromatic OH-groups, including those which do not contribute to the antioxidation. A different trend was observed for ABTS assay compared to DPPH assay (referred to HAE); in fact, the DPPH test provided lower results than those of the ABTS assay, probably because of the different HAE interaction with the radical. The differences between ABTS and DPPH hydrophilic assays performed on HAE may also be due to sample colour. It was determined that the colour of the sample can affect the absorbance values obtained with spectrophotometric assay. Arnao [64] reported that in the DPPH, the problem is more serious than in the ABTS assay since it does not present bands higher than 515 nm. Therefore, at this wavelength, the antioxidant activity measured is underestimated. Moreover, Suarez et al. [65] also determined differences between DPPH and ORAC assays, confirming that the DPPH assay is not suitable for the determination of the antioxidant activity of a matrix characterised by high complexity.

Results from the DPPH assay performed on the extracted oils were higher than those in the hydrophilic fraction (Figure 8c). This was expected, as the DPPH assay in oil also considers tocopherols which show a synergistic action with phenolics [66]. The μmol TE∙100 g^−1^ oil measured by the DPPH assay performed on oil increased from *t0* to *t4*, and then decreased until the end of the storage. This may be due to the formation of melanoidins during baking, which are antioxidant molecules [67,68,69].

### 3.8. Color of the Extracted Oil

**Figure 9:**
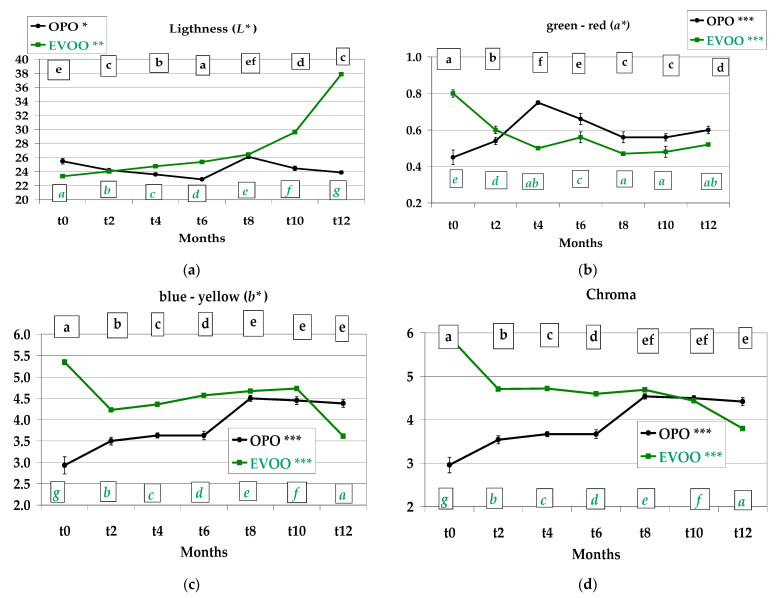
(**a**) Lightness L*, (**b**) green–red a*, (**c**) blue–yellow b*, (**d**) Chroma of olive pomace oil and extra virgin olive oil extracted from *Treccine* during one-year shelf life. The green line and green letters are related to the *Treccine* prepared with EVOO. The black line and black letters are related to the *Treccine* prepared with OPO. Values with different letters with the same colour are significantly different: * *p* < 0.05; ** *p* < 0.01; *** *p* < 0.001.

The lightness of the oil from *Treccine* using the EVOO recipe increased with a constant rate until t8 (from 23.33 to 26.43) and showed a dramatic increase in the last 4 months (37.87 at *t12*). In the oil extracted from the OPO recipe, the values varied significantly ((*p* < 0.01) from 22.90 to 25.47) (Figure 9a). The a* value exhibited an opposite behaviour in the first 4 months of storage with a decrease in the oil of EVOO recipe and an increase in the oil of OPO; after that, the lines designed by data were parallel with oil from OPO recipe, redder than those of EVOO (Figure 9b). The b* value exhibited an opposite behaviour in the first 2 months of storage (OPO increased and EVOO decreased); after that, the a* value increased in both the extracted oils until *t10* and decreased in the last 2 months (Figure 9c). As we determined for the colour of *Treccine*, the behaviour of Chroma was similar to that of b* (Figure 9d). The most remarkable data are the increase in Lightness after 8 months of storage, probably due to the chlorophyll degradation and the consequent loss in colour of EVOO contained in *Treccine*, whereas minor changes were observed in the extracted OPO.

### 3.9. Fatty Acid Composition of the Oil Extracted from Treccine

All values (Table 1, Table 2, Table 3 and Table 4) were within the limits described in the International Regulations [41,42]. In detail, oleic acid varied between 71.08 and 73.28% (*p* < 0.05) in the lipidic fraction obtained from *Treccine* prepared with OPO and varied insignificantly between 72.45 and 73.65% in the lipidic fraction obtained from *Treccine* prepared with EVOO (Table 1 and Table 3). Linoleic acid was higher in the OPO extracted oil than in EVOO *Treccine* recipe. The sum of mono-unsaturated fatty acids prevailed in the oil of the EVOO recipe; reciprocally, the sum of poly-unsaturated fatty acids prevailed in the oil of the OPO recipe (Table 2 and Table 4).

The sum of mono-unsaturated fatty acids increased during storage in the oil extracted from the OPO recipe and reciprocally the sum of poly-unsaturated fatty acids decreased significantly (*p* < 0.05). Non-significant variations were detected in the oil extracted from the EVOO recipe (Table 2 and Table 4). Our results are in agreement with those of Toker et al. [56] who detected a relationship between phenolic content and fatty acid variation during vegetable oil storage: the higher the phenolic content, the lower the fatty acid variation. In vegetable oils with a low phenolic content, they detected a decrease in linoleic acid and an increase in oleic acid, which was due to the two double bonds contained in the linoleic acid and to the higher oxidative attitude of linoleic acid. Edible oils are composed of triacylglycerol molecules, mainly formed by unsaturated and saturated fatty acids esterified to glycerol units [69]. The nutritional value and health functions of virgin olive oil (VOO) are ascribed to the presence of a large amount of monounsaturated fatty acids (MUFAs), such as oleic acid (C18:1), which are the most representative and valuable minor components [70,71,72,73]. Fatty acid composition has a strong influence on the properties of the edible oils and, in particular, on the stability to oxidation. Changes in the fatty acid percentages could occur due to the chemical reactions (oxidation, hydrolysis) as a consequence of heating treatments. In fact, the thermo-lability or volatility of these compounds has great effect [74]. Other authors have studied the fatty acid profiles of different vegetable oils after microwave and/or conventional heating treatments and reported no change or slight and non-significant variations [75,76].

## 4. Conclusions

*Treccine* flavoured with olive and onions were compared in two recipes: the first containing olive pomace oil and the second containing extra virgin olive oil of the Ottobratica cultivar, autochthonous to the Calabria Region, Italy. The physical, chemical and sensory properties of the *Treccine* (evaluated at bi-monthly interval) showed a constant deterioration over time, which was determined to be influenced by the oil used in the recipe. *Treccine* prepared with extra virgin olive oil showed better physical, chemical and sensory properties at each sampling. Water activity and moisture content increased constantly during storage in the *Treccine* of both recipes but at a different rate. Hardness was highest in OPO *Treccine*. L* and b* of *Treccine* showed different behaviours, whereas a* had a very consistent profile during one year of storage. Peroxide value, K232 and K268 in the EVOO recipe were lower than in the OPO recipe throughout storage; in particular, the peroxide value showed a constant increase in OPO from *t0* to *t8* and a dramatic increase from *t8* to *t12*, whereas peroxide value in EVOO remained below 20 meq O_2_/kg oil for almost one year. The colour of the extracted oil was determined to be different in the two different recipes and at the same time was determined to be influenced by the storage time.

The *Treccine* prepared with olive pomace oil were determined to be acceptable to sensory analysis for 4 months of storage, whereas extra virgin olive oil prolonged the acceptability for two more months, which is very important to industries from an organisational point of view. A change in packaging from transparent to non-transparent would improve storage. In addition, using oil from pomace can be considered as an upcycling strategy to reduce food waste, which means it remains an economical option for the food industry.

## Figures and Tables

**Table 1:** Percentage composition of single fatty acids in the extracted lipid fraction of *Treccine* prepared with olive pomace oil. Values with different letters are significantly different: * *p* < 0.05; n.s., not significant.

	14:0	16:0	16:1	17:0	17:1	18:0	18:1	18:2	18:3	20:0	20:1	22:0	24:0
*t0*	0.02	12.03	0.77	0.09	0.14	2.56 ^d^	71.08 ^a^	12.00 ^c^	0.05	0.49	0.43	0.22	0.12
*t2*	0.02	12.06	0.76	0.09	0.12	2.23 ^c^	71.45 ^b^	11.99 ^c^	0.05	0.49	0.41	0.22	0.11
*t4*	0.02	12.35	0.76	0.09	0.12	2.15 ^b^	71.68 ^bc^	11.61 ^b^	0.05	0.48	0.40	0.20	0.09
*t6*	0.02	12.51	0.78	0.09	0.13	2.24 ^c^	71.48 ^b^	11.52 ^b^	0.05	0.47	0.41	0.21	0.09
*t8*	0.01	12.33	0.76	0.07	0.12	1.47 ^a^	72.92 ^d^	11.13 ^b^	0.04	0.47	0.40	0.20	0.08
*t10*	0.02	12.26	0.77	0.09	0.13	1.45 ^a^	73.28 ^e^	10.76 ^a^	0.04	0.50	0.40	0.20	0.09
*t12*	0.02	12.32	0.90	0.11	0.18	1.41 ^a^	72.88 ^d^	10.97 ^a^	0.04	0.48	0.39	0.21	0.09
Sign.	n.s.	n.s.	n.s.	n.s.	n.s.	*	*	*	n.s.	n.s.	n.s.	n.s.	n.s.

**Table 2:** Percentage composition of grouped fatty acids in the extracted lipid fraction of *Treccine* prepared with olive pomace oil. Values with different letters are significantly different: * *p* < 0.05; n.s., not significant.

	Σ Saturated	Σ Unsaturated	Σ Mono -Unsaturated	Σ Poly -Unsaturated	Unsaturated/ Saturated
*t0*	15.51	84.49	72.44 ^a^	12.05 ^c^	5.45
*t2*	15.21	84.79	72.75 ^a^	12.04 ^c^	5.57
*t4*	15.37	84.63	72.97 ^b^	11.66 ^b^	5.51
*t6*	15.61	84.39	72.82 ^ab^	11.57 ^b^	5.41
*t8*	14.62	85.38	74.21 ^c^	11.17 ^ab^	5.84
*t10*	14.60	85.40	74.60 ^d^	10.80 ^a^	5.85
*t12*	14.62	85.38	74.37 ^c^	11.01 ^a^	5.84
Sign.	n.s.	n.s.	*	*	n.s.

**Table 3:** Percentage composition of single fatty acids in the extracted lipid fraction of *Treccine* prepared with extra virgin olive oil. No significant differences were found: *p* > 0.05; n.s., not significant.

	14:0	16:0	16:1	17:0	17:1	18:0	18:1	18:2	18:3	20:0	20:1	22:0	24:0
*t0*	0.01	13.09	1.22	0.15	0.32	2.38	72.60	8.61	0.58	0.48	0.32	0.16	0.08
*t2*	0.01	13.27	1.27	0.15	0.34	2.00	72.45	8.91	0.60	0.48	0.29	0.15	0.08
*t4*	0.01	13.04	1.23	0.15	0.33	1.76	73.43	8.47	0.59	0.46	0.30	0.15	0.08
*t6*	0.01	13.36	1.18	0.18	0.31	1.70	73.65	8.07	0.57	0.45	0.29	0.16	0.08
*t8*	0.01	12.76	1.22	0.16	0.34	2.24	73.07	8.56	0.60	0.48	0.33	0.15	0.08
*t10*	0.01	12.93	1.21	0.15	0.32	2.02	73.44	8.33	0.57	0.47	0.31	0.16	0.08
*t12*	0.01	13.17	1.26	0.17	0.34	2.39	72.64	8.41	0.58	0.48	0.31	0.17	0.08
	n.s.	n.s.	n.s.	n.s.	n.s.	n.s.	n.s.	n.s.	n.s.	n.s.	n.s.	n.s.	n.s.

**Table 4:** Percentage composition of grouped fatty acids in the extracted lipid fraction of *Treccine* prepared with extra virgin olive oil. No significant differences were found: *p* > 0.05; n.s., not significant.

	Σ Saturated	Σ Unsaturated	Σ Mono -Unsaturated	Σ Poly -Unsaturated	Unsaturated/ Saturated
*t0*	16.33	83.67	74.48	9.19	5.12
*t2*	16.13	83.87	74.36	9.51	5.20
*t4*	15.64	84.36	75.30	9.06	5.39
*t6*	15.92	84.09	75.45	8.64	5.28
*t8*	15.87	84.13	74.97	9.16	5.30
*t10*	15.80	84.20	75.30	8.90	5.33
*t12*	16.45	83.56	74.57	8.99	5.08
Sign.	n.s.	n.s.	n.s.	n.s.	n.s.

## Data Availability

The data presented in this study are available on request from the corresponding author.
